# Estimation of microvascular perfusion after esophagectomy: a quantitative model of dynamic fluorescence imaging

**DOI:** 10.1007/s11517-019-01994-z

**Published:** 2019-06-26

**Authors:** Haryadi Prasetya, Sanne M. Jansen, Henk A. Marquering, Ton G. van Leeuwen, Suzanne S. Gisbertz, Daniel M. de Bruin, Ed van Bavel

**Affiliations:** 1 Department of Biomedical Engineering & Physics, Amsterdam University Medical Centers, Amsterdam, The Netherlands; 2 Department of Radiology and Nuclear Medicine, Amsterdam University Medical Centers, Amsterdam, The Netherlands; 3 Department of Surgery, Amsterdam University Medical Centers, Amsterdam, The Netherlands

**Keywords:** Fluorescence imaging, Indocyanine green, Gastric conduit model, Perfusion, Esophagectomy

## Abstract

Most common complications of esophagectomy stem from a perfusion deficiency of the gastric conduit at the anastomosis. Fluorescent tracer imaging allows intraoperative visualization of tissue perfusion. Quantitative assessment of fluorescence dynamics has the potential to identify perfusion deficiency. We developed a perfusion model to analyze the relation between fluorescence dynamics and perfusion deficiency. The model divides the gastric conduit into two well-perfused and two anastomosed sites. Hemodynamics and tracer transport were modeled. We analyzed the value of relative time-to-threshold (RTT) as a predictor of the relative remaining flow (RRF). Intensity thresholds for RTT of 20% to 50% of the maximum fluorescence intensity of the well-perfused site were tested. The relation between RTT and RRF at the anastomosed sites was evaluated over large variations of vascular conductance and volume. The ability of RTT to distinguish between sufficient and impaired perfusion was analyzed using c-statistics. We found that RTT was a valuable estimate for low RRF. The threshold of 20% of the maximum fluorescence intensity provided the best prediction of impaired perfusion on the two anastomosed sites (AUC = 0.89 and 0.86). The presented model showed that for low flows, relative time-to-threshold may be used to estimate perfusion deficiency.

## Introduction

Esophagectomy, despite the arduous nature of the procedure, is a commonly used surgical technique to treat esophageal cancer [1]. The procedure involves gastric transposition to the thorax and removal of major arterial and venous connections. Anastomotic leakage, necrosis, and stricture are major complications of this procedure. The success of esophagectomy depends on maintenance of perfusion of the whole gastric tube [2, 3]. Particularly, in the fundus, i.e., the proximal end of the gastric tube that is anastomosed with the remaining esophagus, perfusion is hampered as in this region, the perfusion fully depends on the presence of collateral connections. Insufficient perfusion hinders anastomotic healing or may even cause tissue necrosis. Early detection of insufficient perfusion could assist clinical decision making on additional surgical intervention, such as determination of the level of the anastomosis and primary anastomotic repair, consequently improving post-operative outcomes [4]. Accordingly, intra-operative monitoring of local perfusion in the gastric tube is needed to predict success of the procedure.

Recent developments in fluorescence imaging (FI) allow intra-operative visualization of local tissue perfusion in the gastric conduit [5–9]. This technique involves intravenous injection of indocyanine green (ICG) and monitoring of its appearance dynamics in gastric tissue. FI has shown a difference in fluorescence dynamics between native and anastomosed areas in the gastric conduit during surgery [4]. Preliminary experiments showed later time-to-peak of contrast arrival, suggesting lower perfusion, in areas closer to the fundus. However, the quantitative relation between contrast dynamics and actual perfusion of the gastric tissue has not been investigated. Moreover, alternative methods for detection of perfusion in this setting are not available.

Impaired perfusion results in slower contrast appearance compared to normal perfusion. Although time-to-peak is commonly used in dynamics measurements to assess perfusion, we hypothesize that time to intensity threshold is a valuable alternative because this measure can be performed in a shorter acquisition window of FI [4]. However, the validity of time to intensity threshold to assess local perfusion is unknown and may be complicated by the complexity of gastric tube vascular network, which includes collateral connections. In this study, we present a comprehensive model that describes the deteriorated perfusion after esophagectomy and its relation with temporal ICG fluorescence intensity profiles. This model was used to explore the relation between reduced perfusion and slower fluorescence enhancement at anastomosed areas. Because the volumes of these vascular compartments affect the dynamics [10, 11], the effect of variations in the vascular architecture of the gastric conduit on the relation between contrast dynamics and local perfusion was studied as well. We included a wide range of vascular resistances and volumes in order to identify general trends in ICG enhancement dynamics as a measure for perfusion. Finally, those trends were used to evaluate the usefulness of time to intensity threshold as an estimate for local perfusion.

## Methods

Essentially, we defined a simulation model of the gastric tube that includes perfusion and ICG transport in four regions. Local perfusion in this model was determined for a large range of model parameters. RRF, the relative remaining flow, is the calculated flow after the intervention relative to the flow to that compartment before the intervention and is considered to be a predictor of clinical outcome. RTT, the relative time to threshold, is the calculated time to a threshold signal for local ICG appearance, normalized to the time to threshold in the first, well-perfused compartment. RTT is considered to be a surrogate for RRF. We analyzed how well RTT predicts RFF, how this depends on the chosen parameters, and which threshold should be taken.

### Gastric conduit model

**Fig. 1:**
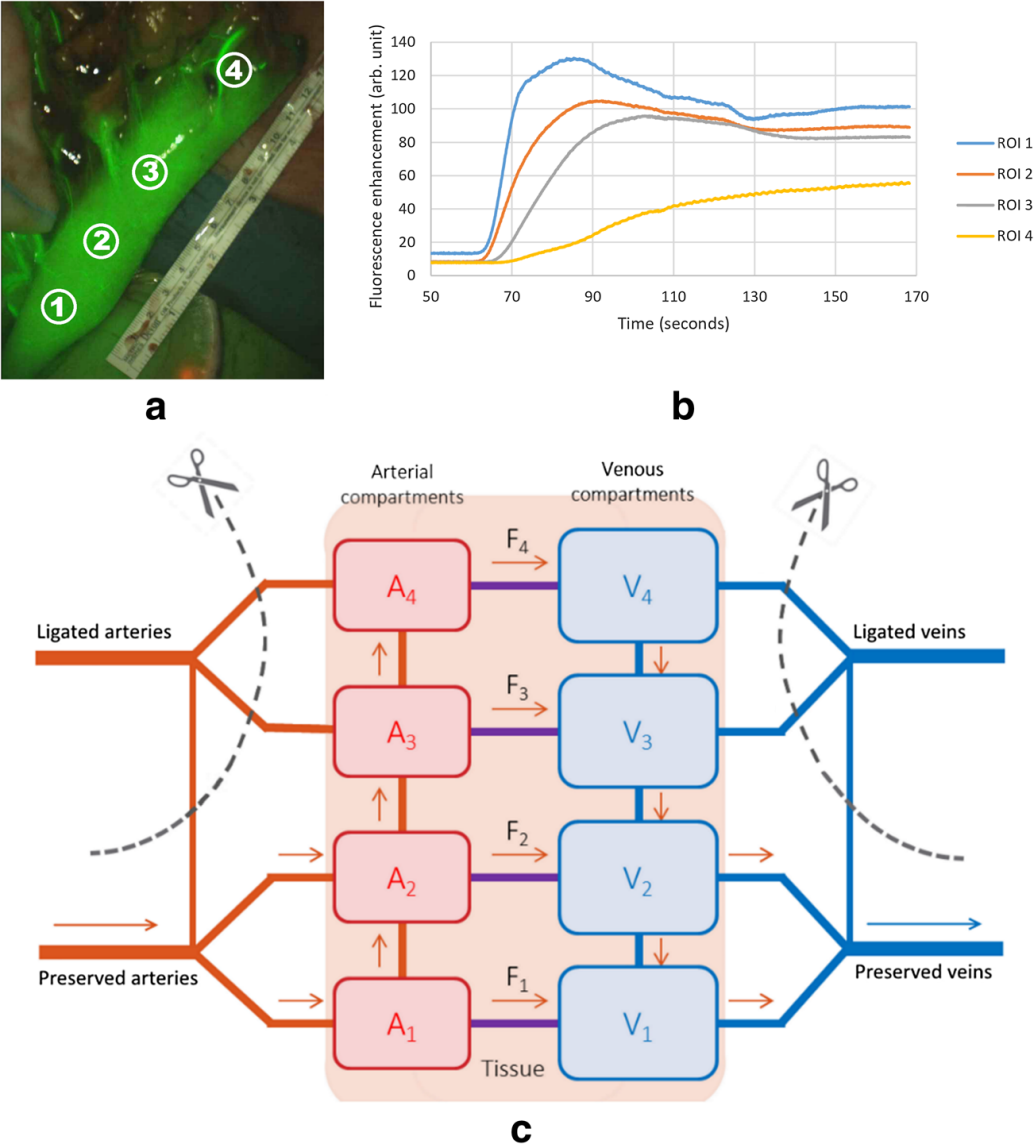
An image of constructed gastric conduit (**a**), obtained intraoperatively during fluorescence imaging post-esophagectomy, demonstrated perfusion with ICG and reduced fluorescence in the collateral-dependent upper part. The corresponding fluorescence enhancement curves of each ROI were measured at the gastric conduit (**b**). The model (**c**): from bottom to top: the sites 1–4, which correspond, respectively, to the ROIs. A site consists of an arterial and a venous compartment directly connected through a capillary bed (horizontal line between compartments). Collaterals, as depicted by vertical lines between compartments, connect the adjacent sites. In this figure, sites 1 and 2 are well perfused, whereas sites 3 and 4 are anastomosed. Arrows indicate direction of flow after the intervention

The reconstruction of the gastric conduit generally preserves the right gastroepiploic vessels and right gastric artery as the main source of blood supply to the gastric wall. Consequently, part of the gastric conduit close to the anastomosis is only supplied with blood from collateral connections in the gastric wall. We modeled the gastric conduit by introducing four sites, as shown in Fig. 1. This choice was based on the four regions of interest (ROIs) for measurement of fluorescence intensity performed on constructed gastric conduit in the prospective clinical study in Amsterdam Medical Center from October 2015 to June 2016 [12]. Figure 1a shows a frame of constructed gastric conduit of a patient with the ICG visualizing the tissue perfusion. The ROI was a 300 pixels circle with #1 3 cm below the watershed, #2 the watershed, #3 3 cm above the watershed, and #4 the fundus. The measured fluorescence enhancement curves at the four ROIS are shown in Fig. 1b. In the model, the four sites are connected through collateral arteries and veins. Site 3 and site 4 represent the anastomosed areas and perfusion here depends completely on collateral vessels. The distributed nature of the arterial, capillary, and venous networks at each site is represented by a single lumped resistance thought to be situated in the arterioles and capillaries, connecting a proximal arterial volume to a distal capillary/venous volume. The vascular bed in the gastric tube is thus represented by eight compartments, four microvascular connections, three arterial collateral connections, and three venous collateral connections. The system is supplied and drained by large arteries and veins of the lower two sites (Fig. 1c).

### Parameter space of the model

We assumed identical vascular conductances and volume for each site and varied multiple model parameters including collateral artery and vein conductance relative to microvascular conductance (*G*_*ca*_/*G*_*c*_ and *G*_*cv*_/*G*_*c*_), relative large vessel conductance (*G*_*LV*_/*G*_*c*_), and vascular volume including arterial and venous volume (*AV* and *VV*). Quantitative information on gastric vascular branching patterns is not available, but we considered that the above conductance ratios are likely to be highly variable between patients. We therefore evaluated the model over a large parameter space, including the presumed physiological range. Determining the physiological range for vessel conductance is difficult. Total conductance of the arterial and venous system depends on the network connectivity and the diameter of the individual segments in this network. Such data are, to the best of our knowledge, not available for the human stomach. We therefore covered a very wide range of values for *G*_*ca*_/*G*_*c*_ and *G*_*cv*_/*G*_*c*_, spanning 0.01 to 100 for both ratios. The low end of the chosen spectrum reflects absence of collaterals, while the high end indicates a model with identical perfusion of all four sites. Physiological values were estimated to be less than 10, with no definitive lower boundary [13, 14]. The large vessels (LV) include terminal arteries, i.e., right gastric artery and right gastroepiploic terminal artery, and initial veins, i.e., pyloric vein and right gastroepiploic initial vein. Relative conductance of the combined large arteries (*G*_*LV*_/*G*_*c*_) was 3 by default and spanned 1 to 100 when studying the effect of this parameter. Large vein conductance was fixed at twice the large artery conductance. We used physiological data describing the proportion of artery, capillary, and vein in a given volume in the systemic circulation to determine the physiological vascular volumes [14, 15]. The total volume of a site was calculated based on the volume of the ROI in the preceding clinical study [12]. The default vascular volumes were taken as 0.24 mL and 0.56 mL for arterial (*AV*) and venous (*VV*) compartments, respectively, spanning 0.12 mL to 1.12 mL when studying the effect of vascular volume to contrast dynamics.

### ICG transport simulation

Pressure at each branching point in Fig. 1c and flow in each segment was calculated using Kirchhoff’s first law combined with Ohm’s law, assuming laminar flow of a Newtonian fluid [16]. These calculated hemodynamic parameters depend on the pressures in the right gastroepiploic artery and vein and on the conductance of all segments. Arterial input and venous outflow pressure were taken as 70 mmHg and 0, respectively. Conductances were varied to evaluate their influence on perfusion. For each segment, after the pressure gradient was obtained, the flow was calculated. Tissue perfusion at the four sites is reflected by the predicted flow in the segments connecting arterial and venous compartments (*F*_1_ to *F*_4_).

The ICG enters the system from the hepatic artery into the gastroduodenal artery (greater curvature), which leads into right gastroepiploic artery, and the right gastric artery (lesser curvature). The ICG then flows into the various compartments from *A*_*1*_ and *A*_*2*_ towards capillaries in the native sites and collateral-dependent sites and leaves the system via *V*_*1*_ and *V*_*2*_. ICG from the right gastric and the right gastroepiploic veins drains into the portal and the superior mesenteric vein, respectively. This ICG transport was simulated using the above vessel configuration and well-mixed arterial and venous compartments, where dynamics of dye concentration obey the following differential equation:

1 $$ \frac{d{C}_k}{dt}=\frac{\sum \limits_{j=1}^mU{C}_j\cdot U{F}_j-{C}_k\sum \limits_{i=1}^nD{F}_i}{V_k} $$

with *C*_*k*_ the concentration of the dye in compartment *k*, *m*, and *n* the number of upstream and downstream vessel of compartment *k*, respectively, *UC*_*j*_ the concentration of upstream compartment j, *UF*_*j*_ upstream flow from this compartment, *DF*_*i*_ flow through *i*-th downstream vessel, and *V*_*k*_ the volume of compartment *k* [17]. This ordinary differential equation was numerically solved with a single-step solver based on the Dormand-Prince algorithm of Runge-Kutta method which computed *C(t)* from [*C(t-Δt)*] [18]. The algorithm had six stages of function evaluation for each partial step and generated fourth-order and fifth-order approximation of *C(t)*. The two approximations was subsequently compared to estimate the error which provides the basis to accept or reject the tentative *C(t)*. The error estimate also modulated the step size, *Δt*, for the next time step.

**Fig. 2:**
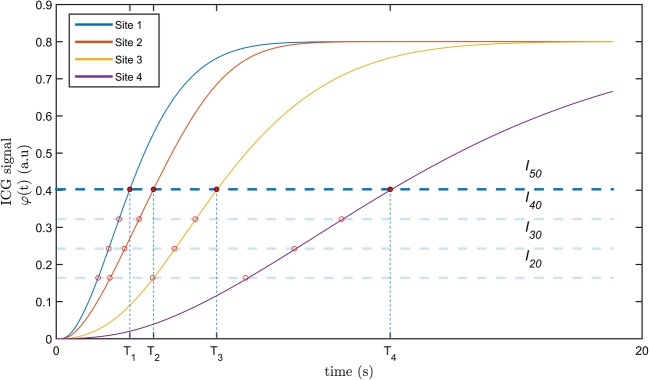
Example of contrast dynamics at the four sites with derivation of the respective times to threshold. Sites 1 and 2 have native perfusion, while sites 3 and 4 depend on collateral flow, and accordingly have slower contrast dynamics

The ICG signal intensity for each site was defined as the amount of ICG in both the arterial and venous compartments at time *t*:

2 $$ {\varphi}_i(t)=A{C}_i(t)\cdot A{V}_i+V{C}_i(t)\cdot V{V}_i $$

where *φ* indicates the ICG signal, *AC* and *AV* denote the ICG concentration and the volume of the arterial compartment, respectively. *VC* and *VV* represent the ICG concentration and the volume of the venous compartment and *i* denotes the number of site. Figure 2 shows an example of the simulated temporal profile of *φ* for each site.

### Time-based perfusion estimate

For each simulation, the temporal profile of *φ* at each site was analyzed. This signal forms the base for the estimation of remaining perfusion after surgery. We propose a measure to describe relative impairment of flow in a collateral-dependent site. This measure was based on the time to threshold *T*_*n*_ which was defined as the time at which *φ* of site *n* reaches a fraction of the maximum of *φ* of site 1. For example, in Fig. 2, *T*_4_ is defined as the time it takes for *φ* to reach 50% of the maximum intensity in site 1 (*I*_50_). This approach ensures that *T*_n_ can be calculated as long as the maximum *φ* in site 1 is recorded. We introduce RTT as a parameter on which RRF estimation can be based:

3 $$ {\displaystyle \begin{array}{l} RT{T}_3={T}_1/{T}_3\\ {} RR{F}_3={F}_3/{F}_{3 presurgery}\end{array}}\kern0.5em {\displaystyle \begin{array}{l} RT{T}_4={T}_1/{T}_4\\ {} RR{F}_4={F}_4/{F}_{4 presurgery}\end{array}} $$

With *T*_*i*_ the time to threshold and *F*_*i*_ the flow in site *i*. *F*_*presurgery*_ is the flow prior to the ligation. It was calculated from the same model and using the same set of parameter values but with *A*_3_ and *A*_4_ still supplied by the large arteries and *V*_3_ and *V*_4_ still drained by the large veins. A range of intensity thresholds were tested: 20%, 30%, 40%, and 50% of the maximum *φ* of site 1. The starting point (*t* = 0) was defined as the first inflection of contrast intensity in site 1. RRF is the ratio of post- and pre-intervention flow. We hypothesized that for a large range of variation in vascular conductances and volumes, RTT is closely related to RRF, such that RTT is a possible measure for the effect of surgery on local perfusion.

Using the simulation scenarios detailed in the Table 1, we first evaluated the effect of *G*_*ca*_/*G*_*c*_ and *G*_*cv*_/*G*_*c*_ on the remaining perfusion in anastomosed sites by fixing *G*_*LV*_/*G*_*c*_, *AV* and *VV* to their default values (simulation 1). The effect of *G*_*LV*_/*G*_*c*_ was tested while fixing *AV* and *VV* to 0.24 and 0.56 ml, respectively (simulation 2). Finally, *G*_*LV*_/*G*_*c*_ was maintained at its default value (3) while examining the effect of *AV* and *VV* (simulation 3). All tests were performed using all intensity thresholds over the full range of collateral conductances. We then computed the RRF for all possible combinations of parameters for a range of possible outcomes (sample space) from Ohm’s and Kirchhoff’s laws. We included curve fits of the relation between RRF and RTT to illustrate the nature of the relation over collateral conductance space. We included curve fits of the relation between RRF and RTT to illustrate the nature of the relation over collateral conductance space. We tested the curve fit using linear, polynomial, and exponential function while evaluating the goodness-of-fit. A good fit was defined as a model that has low sum of squared of errors and high *R*^2^. The prediction interval was calculated by taking into account the sample mean, sample standard deviation, sample size, and critical value of Student’s *t* distribution at 95% confidence level. It should be noted that the indicated relations merely described the data and were not a result of mathematical analysis of the model. Hence, these fits do not affect the further outcomes in this study. Finally, we performed receiver operating characteristic (ROC) curve analysis to select the best intensity threshold to estimate perfusion impairment. We arbitrarily chose RRF values to dichotomize the outcome into lacking versus adequate perfusion. For site 3, RRF < 50% was defined as lacking perfusion, and RRF ≥ 50% was adequate perfusion. For site 4, RRF < 40% was defined as lacking perfusion, and RRF ≥ 40% was adequate perfusion. The area of physiological sample space was treated as predictor variables on which logistic regression was applied to produce the ROC curve. The simulation was performed in Matlab on a standard PC running on Windows 7 with 3 GHz 16 CPUs, 32 GB RAM, and NVIDIA Quadro K4200 GPU.

**Table 1:** Parameters of the model, simulation scenarios, and generated variables

Parameters	Definitions [unit]	Default	Min	Max
*G*_*ca*_/*G*_*c*_	relative collateral artery conductance [−]		0.01	100
*G*_*cv*_/*G*_*c*_	relative collateral vein conductance [−]		0.01	100
*G*_*LV*_/*G*_*c*_	relative large vessel conductance [−]	3	1	100
*AV*	arterial volume [ml]	0.24	0.12	1.12
*VV*	venous volume [ml]	0.56	0.12	1.12
Simulation 1				
Model parameters varied: *G*_*ca*_/*G*_*c*_, *G*_*cv*_/*G*_*c*_				
Model parameters fixed to default: *G*_*LV*_/*G*_*c*_, *AV*, *VV*				
Simulation 2				
Model parameters varied: *G*_*ca*_/*G*_*c*_, *G*_*cv*_/*G*_*c*_, *G*_*LV*_/*G*_*c*_				
Model parameters fixed to default: *AV*, *VV*				
Simulation 3				
Model parameters varied: *G*_*ca*_/*G*_*c*_, *G*_*cv*_/*G*_*c*_, *AV*, *VV*				
Model parameters fixed to default: *G*_*LV*_/*G*_*c*_				
Generated variables				
Solving Kirchhoff’s first law + Ohm’s law	*F*_1_, *F*_2_, *F*_3_, *F*_4_			
	*F*_presurgery_*			
Solving eq.1 & eq.2	*T*_1_, *T*_2_, *T*_3_, *T*_4_; for threshold 20% of maximum *φ* of site 1 (*I*_20_)			
	*T*_1_, *T*_2_, *T*_3_, *T*_4_; for threshold 30% of maximum *φ* of site 1 (*I*_30_)			
	*T*_1_, *T*_2_, *T*_3_, *T*_4_; for threshold 40% of maximum *φ* of site 1 (*I*_40_)			
	*T*_1_, *T*_2_, *T*_3_, *T*_4_; for threshold 50% of maximum *φ* of site 1 (*I*_50_)			

^*^ *F*_*presurgery*_ *were similar across the sites*

## Results

**Fig. 3:**
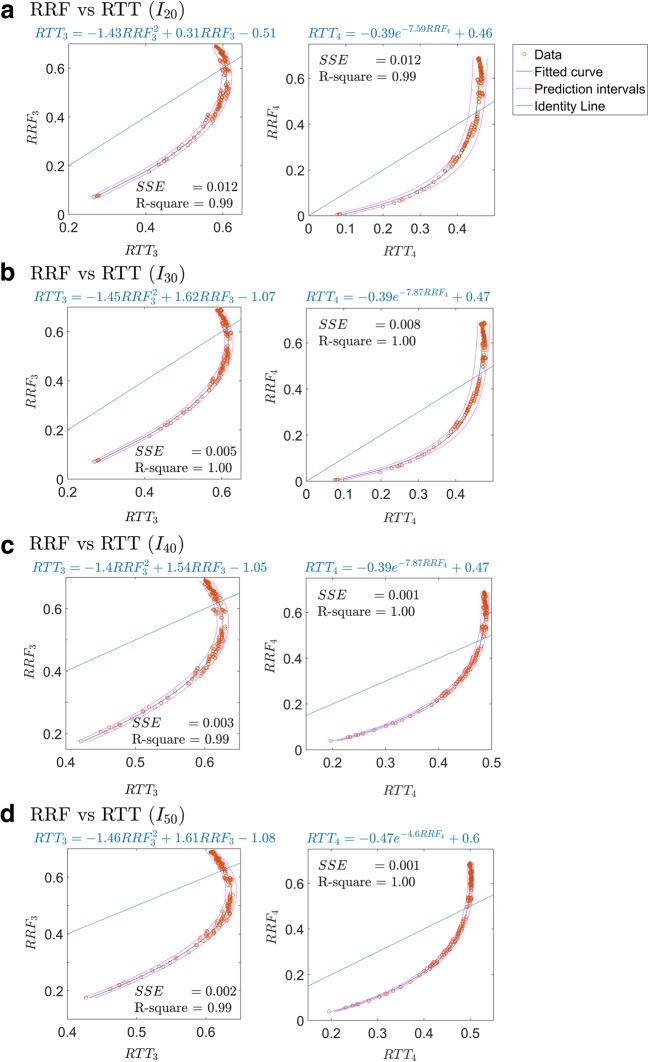
Result of simulation 1: relation between RTT and RRF over collateral conductance space, for sites 3 (left) and 4 (right). The plots show RTT for thresholds of contrast arrival at 20% (**a**), 30% (**b**), 40% (**c**), and 50% (**d**) of the maximum contrast signal at site 1 as a function of RRF. The corresponded fitting functions and the respective goodness-of-fit parameters were also displayed

**Fig. 4:**
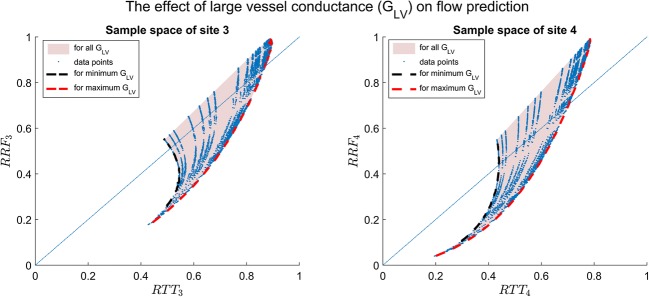
Result of simulation 2: variation in large vessels conductance changes the relation between true RRF and its estimate (RTT)

**Fig. 5:**
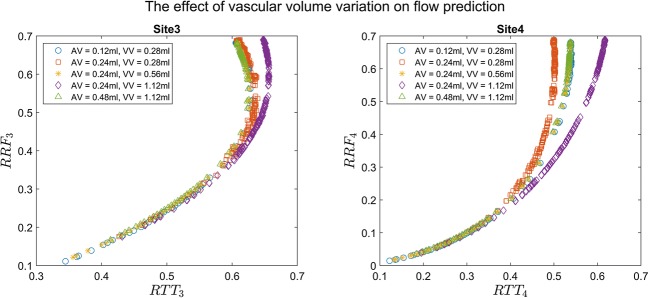
Result of simulation 3: RRF and RTT relation as a function of the ratio of arterial volume (*AV*) and venous volume (*VV*). Note that not every symbol/color is visible due to the overlay

The relation between RRF and RTT for sites 3 and 4 is shown in Fig. 3, where the estimates are based on time to reach 20% (Fig. 3a) to 50% (Fig. 3d) of the maximum *φ*. The increase in collateral conductance correlates with higher RTT and RRF. Interestingly, aside from negligible variation in the resulting RRF, the varying balance of conductance between collateral arteries and collateral veins seems to be inconsequential (see Appendix). As can be seen, the relation between RTT and RRF is far from linear, with RRF being lower than RTT for low flows. For high flows, RTT becomes stable and insensitive to flow changes. We obtained good fits of the relation at site 3 by second-order polynomials, while for site 4, an exponential fit was needed. The RTT-RRF relation for various values of *G*_*LV*_ is illustrated in Fig. 4. For low values of *G*_*LV*_, the large vessels limit perfusion, thereby reducing the maximum possible levels of RRF. Figure 5 shows the RTT-RRF relation for various vascular volumes. The RTT-RRF relation for low flows is relatively independent of the volumes. However, for higher flows, i.e., better collaterals, the volumes indeed have an influence but rather than the absolute volume values, it is the ratio of arterial and venous volume that matters.

**Fig. 6:**
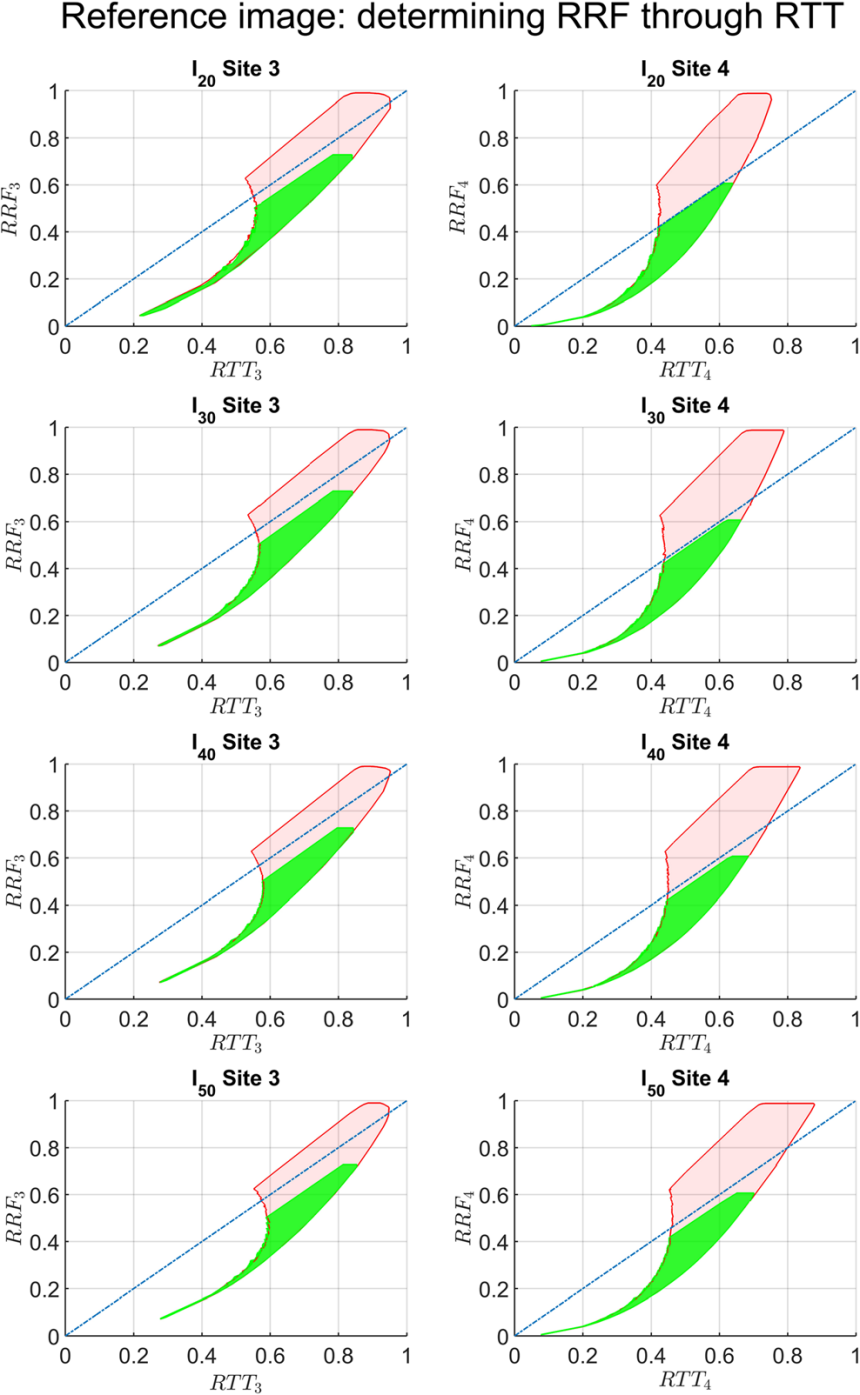
The sample space of the model from the complete (pink) and physiological (green) parameter space of collateral conductances (*G*_*ca*_, *G*_*cv*_), large vessel conductance (*G*_*LV*_), and vascular volume (*AV*, *VV*) was calculated from *I*_*20*_ to *I*_*50*_ (top to bottom)

Figure 6 shows the relation between RTT and RRF for all above variations in conductances and volumes (4 sets of parameters). The pink area indicates the sample space for the full range of variations, while the green area denotes possible outcomes when the parameters were varied between more realistic values.

**Fig. 7:**
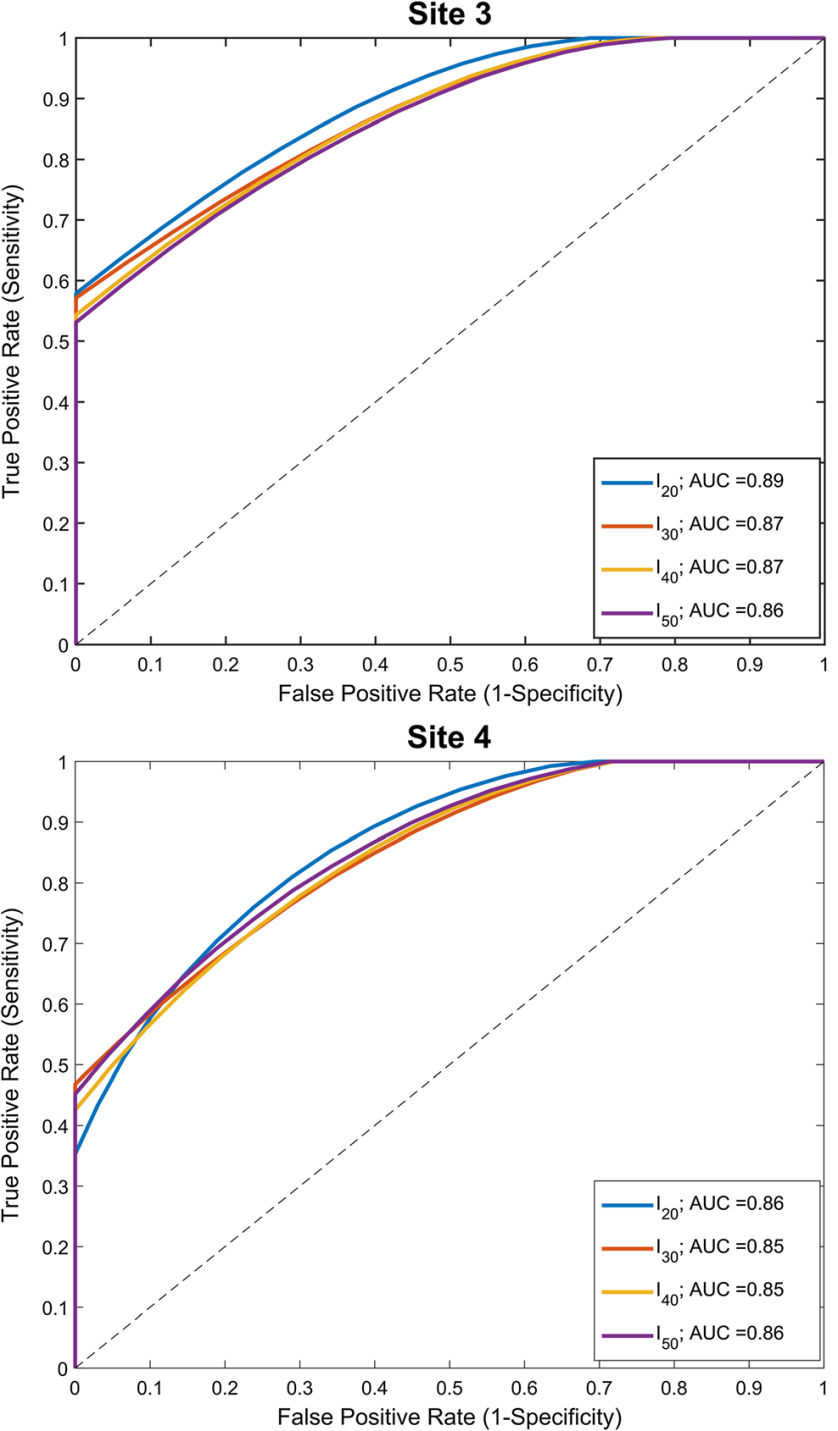
ROC curve of four threshold for site 3 (top) and 4 (bottom). The cutoff value was determined hypothetically as 0.5 and 0.4 for sites 3 and 4, respectively

The highest accuracy of RTT as RRF estimator resides in both extrema of RTT, particularly in the lower values. As example, for an observed RTT with *I*_*20*_ at site 4 of 0.2, the true RRF may have been 0.038 to 0.04, as compared to RTT of 0.5 which may have resulted from a larger range of true RRF from 0.31 to 0.5. Assessed by ROC, all four logistic regression models demonstrated good discriminatory capacity in both sites (see Fig. 7). Although all intensity thresholds performed comparably well, *I*_*20*_ yielded the highest concordance of predictions with actual outcomes (AUC = 0.89 and 0.86 for sites 3 and 4, respectively).

## Discussion

In this study, we introduced a gastric conduit model and used this to identify the effects of various combinations of vascular conductance and volume on ICG dynamics. The results indicate that the relation between delayed time-to-threshold (indicated by RTT) and reduced flow (indicated by RRF) is not trivial. We have shown a strong dependency of this relation on collateral conductance, large vessel conductance, and vascular volume. Despite this dependency, we found that time-to-threshold at the collateral-dependent sites can be used to estimate whether remaining perfusion is sufficient.

### Perfusion model analysis

The vascular tree can be modeled using several choices for the level of detail of the branching network. One extreme is to have a full network of vessel segments, with poroelastic models for the smallest vessels [19–21]. The other extreme would be a single-vessel lumped model that disregards the detailed geometric structure of vascular tree thus reducing the complexity of the model [10, 22, 23]. We believe that the current choice for a limited set of compartments is optimal for the current purpose since the actual small vessel structure in the ROI of the gastric conduit is not well-defined and likely to be highly variable. The four ROIs used in this study, which account for both arterial and venous volumes, are based on a preliminary study using clinical data [12]. The ROI 1, 2, and 3 were equidistant (3 cm apart), while ROI 4 was located in the fundus. We separated arterial and venous network into two compartments of lumped vessels since these compartments influence the contrast dynamics in each ROI. Hence, this model allowed us to evaluate the role of the arterial and venous collateral on the tissue perfusion. Other configuration of the model was not investigated (for instance an eight-site model). This choice would affect the values of the modeling parameters, since clearly the volumes and conductances in each site would be different. Yet, there seems little reason to suspect that the modeling outcomes and further analysis would be fundamentally different for such a more detailed model. Considering the clinical setting we therefore used the four site model.

### Limitations

The validity of the presented model remains a limitation as this study only uses hypothetical data. We partly addressed this limitation by varying several model parameters over large ranges. Yet, many other choices would have been possible. Thus, the assumption of identical collateral conductances for all sites is an oversimplification of real cases. Patient data also show high variability in vascular volume between sites. These variations may yield different relations between RTT and RRF. The accuracy of the predictive value of RTT to predict RRF was based on perfusion thresholds of 50% and 40% of the pre-intervention perfusion. Currently, we do not have data for realistic values of this threshold.

A strong point of this study is that we have created a model in which many parameters can be tested. Therefore, alternative measures (such as intensity based, or absolute times) for estimating the perfusion can be evaluated with this model. However, in the context of the complex surgery, observation periods are limited to 2–3 min and longer periods are a concern [24]. In the fluorescence enhancement curve in Fig. 1b, the fluorescence yield is reasonably similar between the sites and a maximum is obtained in also the fourth site but these are not always the case. Thus, alternative measures of perfusion that rely on normalization to the maximal fluorescence at site 4 or generally the full enhancement curve may have been impractical.

### Potential use of gastric conduit model

A large number of studies have employed fluorescence imaging in intraoperative applications to assist visualization of blood flow or anatomical features [6, 25–28]. However, quantitative analysis of ICG fluorescence imaging of the gastric conduit is still limited. Yukaya et al. introduced a quantitative parameter describing the decay of luminance as analyzed with the software tool LumiView to predict anastomotic leakage [29]. They could not find an association between blood flow and anastomotic leakage. A study on quantitative assessment of free jejunal graft used the time-fluorescence intensity curve, showing that time to half maximum is an indicative parameter for venous malperfusion [30]. However, that study had a population of only five patients suffering from venous anastomotic failure. Furthermore, in that study, no direct relation between perfusion deficit and ICG intensity dynamics was studied. A more recent study had been performed to predict anastomotic leakage by quantitatively measuring ICG speed between four predetermined points in the gastric conduit [8]. Also, this study suffered from limited data especially in the anastomotic leakage/malperfusion group.

While there clearly are several limitations to consider, quantitative analysis of contrast dynamics could provide a useful prognostic tool in determining treatment success. We found that time to 20% of the maximum intensity is optimal in the discrimination between intermediate and low perfusion as indicated by the area under the ROC curve. Additionally, if adopted in clinical practice, this low threshold requires only a relatively short measurement time of fluorescence imaging after maximum intensity is reached in ROI 1, which alleviates surgery-related risks.

Since fluorescence imaging allows assessment of temporal ICG intensity for different areas of gastric conduit intraoperatively, the operating clinician can evaluate intensity profiles for selected ROIs. This allows the calculation of the RTT at the anastomosed site and this value can be used to estimate the range of perfusion reduction (Fig. 6). The actual usefulness of RTT as an estimate for local perfusion and predictor of final outcome in esophagectomy remains to be established.

## Conclusion

Our model demonstrated the effects of vascular conductance and volume on contrast dynamics in gastric conduit in relation to perfusion in anastomosed areas. After evaluating ICG dynamics for numerous different model parameters, we found that the relation between the dynamics and perfusion is not trivial. However, the model showed that for low flows, a low time to threshold intensity is predictive of flow deterioration. This estimation of remaining perfusion may form the base for clinical evaluation of a successful esophagectomy.

## Figures and Tables

**Fig. 8:**
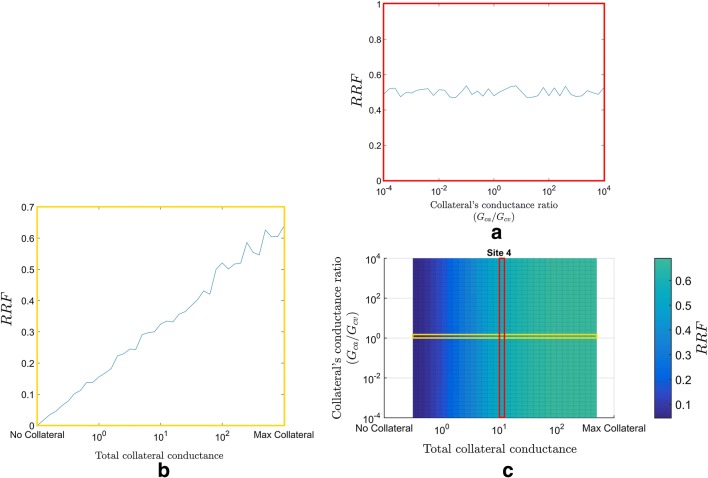
The effects of the balance of arterial and venous collateral conductance (a) and of the total collateral conductance (b) on the relative remaining flow (RRF) in the anastomotic site 4, demonstrating the log-linear dependence of RRF on total collateral conductance without an effect of arteriovenous balance. RRF for all levels of total collateral conductance and arteriovenous balance (c), demonstrating absence of interactive effects of both parameters

## Appendix

*The effect of collateral artery and vein conductance ratio and total collateral conductance to capillary perfusion*

The balance ratio between collateral artery and vein conductance (*G*_*ca*_/*G*_*cv*_) reflects the contribution of one side of collateral over another in determining perfusion. We compared the RRF modeled with a specific collateral conductance ratio with its inverse to test whether a certain side of collaterals is more favorable for perfusion. The result has shown negligible difference between RRF of two conductance configuration. Eventually, it was apparent that the total collateral conductance is a more consequential factor in determining the portion of contrast flowing through the capillary. Total collateral conductance was calculated as the inverse of the sum of collateral arteries and veins resistance in a series circuit (*G*_*ca*_*·G*_*cv*_/(*G*_*ca*_ + *G*_*cv*_)). Figure 8 shows the effect of this total collateral conductance in logarithmic scale on relative remaining flow in these models. The ratio of arterial and venous conductance has a relatively minor impact on these perfusions while increasing total collateral conductances show a better perfusion of the collateral-dependent sites.
